# Contact between Doctors and the Pharmaceutical Industry, Their Perceptions, and the Effects on Prescribing Habits

**DOI:** 10.1371/journal.pone.0110130

**Published:** 2014-10-16

**Authors:** Klaus Lieb, Armin Scheurich

**Affiliations:** Department of Psychiatry and Psychotherapy, University Medical Centre Mainz, Mainz, Germany; York University, Canada

## Abstract

**Background:**

The prescribing behaviour of doctors is influenced by the pharmaceutical industry. This study investigated the extent to which contacts with pharmaceutical sales representatives (PSR) and the perception of these contacts influence prescribing habits.

**Method:**

An online questionnaire regarding contact with PSRs and perceptions of this contact was sent to 1,388 doctors, 11.5% (n = 160) of whom completed the survey. Individual prescribing data over a year (number of prescriptions, expenditure, and daily doses) for all on-patent branded, off-patent branded, and generic drugs were obtained from the Bavarian Association of Statutory Health Insurance Physicians.

**Results:**

84% of the doctors saw PSR at least once a week, and 14% daily. 69% accepted drug samples, 39% accepted stationery and 37% took part in sponsored continuing medical education (CME) frequently. 5 physicians (3%) accepted no benefits at all. 43% of doctors believed that they received adequate and accurate information from PSRs frequently or always and 42% believed that their prescribing habits were influenced by PSR visits occasionally or frequently. Practices that saw PSRs frequently had significantly higher total prescriptions and total daily doses (but not expenditure) than practices that were less frequently visited. Doctors who believed that they received accurate information from PSRs showed higher expenditures on off-patent branded drugs (thus available as generics) and a lower proportion of generics. The eschewal of sponsored CME was associated with a lower proportion of on patent-branded drug prescriptions, lower expenditure on off-patent branded drug prescriptions and a higher proportion of generics. Acceptance of office stationery was associated with higher daily doses.

**Conclusions:**

Avoidance of industry-sponsored CME is associated with more rational prescribing habits. Furthermore, gift acceptance and the belief that one is receiving adequate information from a PSR are associated with changed prescribing habits. Further studies with larger sample sizes are needed.

## Introduction

The prescribing behaviour of doctors is influenced by a number of factors, one of which being visits from the pharmaceutical sales representatives (PSR) of pharmaceutical companies. It is estimated that approximately 15,000 PSRs carry out some 20 million visits to medical practices and hospitals in Germany every year [1]. During these visits, the PSRs inform the doctors about their company's products and new publications, and they use a variety of marketing strategies to motivate the doctors to prescribe their products. In an earlier study, we were able to demonstrate that 77% of doctors in Germany were visited by PSRs at least once a week, and 19% of doctors were visited daily. We showed that drug samples, stationery and dinner invitations were the most frequently accepted gifts [2]. We were also able to show that 53% of doctors believed that their prescribing habits were occasionally, frequently or always influenced by PSRs, while 45% believed that this was rarely or never the case. Based on these subjective assessments by the doctors, we can assume that PSRs have at least a certain degree of influence on medical prescribing habits. Whether PSR visits and the perceptions or subjective opinions of the doctors regarding these visits have an impact on objective prescribing data is not yet known for Germany. With a few exceptions, studies from other countries, such as the seminal study by Avorn et al. [3] and the study by Becker et al. [4] have shown that contact between doctors and pharmaceutical companies is associated with more frequent prescriptions, higher expenditure and lower prescription quality (for a systematic review see [5]). The objective of this study was to use a questionnaire sent to German GPs and practice-based specialists to record the types of contact and their perceptions of these contacts and to determine what effects doctor-PSR contact and the doctors’ perceptions of these contacts might have on prescribing habits. The key features of this study are that 1) the impact on the prescribing data for various product categories, i.e. on-patent and off-patent branded drugs as well as generics, was analysed in detail, and 2) prescribing behaviour was analysed not only in connection with the frequency of PSR visits, but also in connection with the doctors’ subjective opinions with regard to the adequacy of the information received and the extent to which they felt that they had been influenced.

## Method

From a pool of approximately 20,000 prescribing doctors covered by the Bavarian Association of Statutory Health Insurance Physicians, the mean prescription volume from the first three quarters of 2010 was used to form a sub-group of 368 psychiatrists or neurologists with a prescription volume of >€100,000 per quarter, 1,826 GPs or internal medicine specialists working in general practice with a prescription volume of >€100,000 per quarter, and 75 cardiologists with a prescription volume of >€20,000 per quarter (by limiting the prescription volumes to the German average, we sought to exclude physicians with very low prescription volumes). From this total of 2,269 doctors, 1,386 had an email address registered with the Bavarian Association of Statutory Health Insurance Physicians, and these were contacted by email. There were no current funding sources for this study, and the doctors did not receive any remuneration for their participation in the study. As in our previous study [2], we did not ask the local ethics committee for approval of the study, as there was no patients participation and it was only health professionals who answered questions about their contacts with the pharmaceutical industry.

The online questionnaire went live on 24.06.2011 and initially ran until 05.08.2011. The questionnaire was then extended until 31.10.2011 in order to increase the response rate. One reminder was sent to those physicians who had not yet participated after 3 months although no further reminders were sent out to those who had expressed themselves negatively about the questionnaire.

The doctors completed the following online questionnaire about the frequency of PSR visits over the previous year and about their attitudes to them:

1. How often have you been visited by pharmaceutical sales representatives during the last 12 months? (daily, 2–3/week, 1/week, 2/month, occasionally, never)
2. If your response to question 1 was “never”: Why did you not have any contact with pharmaceutical sales representatives?
3. I receive adequate and accurate information about new drugs and therapies from pharmaceutical sales representatives (always, frequently, occasionally, rarely, never)
4. Is your prescribing behaviour being influenced by pharmaceutical sales representatives? (always, frequently, occasionally, rarely, never)
5. Did you give interviews to pharmaceutical sales representatives regarding your prescribing behavior or new therapies, for which you were paid? (always, frequently, occasionally, rarely, never)
6. Which gifts offered by pharmaceutical sales representatives have you accepted during the last 12 months? Office stationery (e.g. ballpoint pens, notes and memos), day-to-day items (e.g. coffee cups, diaries, calendars), drug samples, dinner invitations, sponsored CME events, others (always, frequently, occasionally, rarely, never).

These data were then examined in connection with the prescribing habits of those doctors and assessed anonymously.

The prescribing data for the doctors for the period covered by the questionnaire (namely from the start of the 3rd quarter of 2010 to the end of the 2nd quarter of 2011) were collated and presented in the following categories:

– Expenditure, number of prescriptions, defined daily dose (DDD, i.e. the mean daily dose of a given drug), number of patients per participant for the individual quarters and for the period as a whole
– Expenditure, number of prescriptions, DDD for the individual quarters and for the period as a whole, per participant and manufacturer
– Expenditure, number of prescriptions, DDD for on-patent branded drugs, off-patent branded drugs, and generics.

The following parameters were also calculated: the proportion of generic use for drugs where a generic is available (proportion of generics) = Expenditure on generics×100/(Expenditure on off-patent branded drugs + Expenditure on generics), the proportion of on-patent branded drugs = Expenditure on on-patent branded drugs×100/(Expenditure on all drugs) and the proportion of off-patent branded drugs = Expenditure on off-patent branded drugs×100/(Expenditure on all drugs).

In most cases, the results were summarised as a percentage value and as absolute figures. Statistical analysis was performed using a t-test. For non-normal distribution data, the Mann-Whitney U test was used. All p-values were two-sided. This was an explorative study of potentially influential factors on the behaviour of the doctors questioned. In particular, the following questions were analysed: Are prescription volumes different for doctors receiving a different frequency of visits from PSRs and/or for doctors who accept gifts and drug samples? What is the relationship between the level of influence felt and the prescription volume? As the questions about the visits from the pharmaceutical companies were analysed for differences in terms of expenditure, number of prescriptions and DDD in the categories of on-patent and off-patent branded drugs and generics, there were up to 14 comparisons per question. We aimed at avoiding undue inflation of a type I error due to multiple testing by setting the nominal level of significance at 0.005. This is not as rigid as a Bonferroni adjustment, but we expect that the (actual) multiple type I error does not exceed 0.05 by far, because the variables on pharmaceutical contacts and the variables characterizing prescribing habits, are highly interrelated, respectively. This procedure is based on that set out by Darlington (1990) and takes into consideration multiple tests for associated hypotheses [6]. Effect sizes (Cohen’s d) were calculated for significant results taking into account different sample sizes. For the processing of data and statistical analyses, SPSS (Statistical Package for the Social Sciences Program) for Windows 21.0 was used.

## Results

### Sample

Of the 1,386 doctors contacted, 11.5% (n = 160) completed the online questionnaire. These included 131 GPs/internal medicine specialists, 26 psychiatrists or neurologists and 3 cardiologists. 88% (n = 141) of the participants were men and 12% (n = 19) were women. The mean age was 57.3 years (SD = 7.5); 29% (n = 47) were aged 40–49 years and 42% (n = 67) were aged 50–59 years.

In order to test for sampling bias, the study group of 160 participating doctors was compared to the 1,226 doctors who were contacted, but did not answer. With a mean age of 53.7 years, the participants were on average slightly older than the non-participants (mean age = 52.3 (SD = 8.0) years, p<0.05) (mean age of all primary care physicians in Bavaria: 52 years, and of all psychiatrists and neurologists: 51.5 years). With respect to gender, the group of non-participating physicians consisted of 1042 men and 184 women (Chi^2^, p = 0.342) (gender distribution in primary care physicians in Bavaria 2010: 33.2% women: 66.8% men; [7]). In terms of costs per prescription, there was also no difference between the groups (€60.9 vs. €58.9, p = 0.78).

### Nature of the contact with the PSRs

According to their own estimates, 84% (n = 135) of the doctors were visited at least once a week by PSRs and 14.6% (n = 23) were visited daily. 48.7% (n = 77) were visited 2–3 times per week, 22.2% (n = 35) once weekly, 3.8% (n = 6) twice per month, 5.1% (n = 8) occasionally, and 5.7% (n = 9) never. Free-text responses as to the reasons for not accepting visits from PSRs were (10 responses): “pure advertising”, “no-show practice”, “they are useless”, “I am a member of MEZIS e.V. (German branch of the “No free lunch organization”; www.mezis.de)”.

According to their own estimations, drug samples, office stationery and sponsored CME were the most frequently accepted benefits (see Table 1). According to the information provided by the doctors, 5.7% (n = 9) never accepted drugs samples, 5.7% (n = 9) never accepted office stationery and 39.5% (n = 62) never took part in sponsored CME activities. In total, only 3% (n = 5) of those questioned accepted no benefits at all. 69% (n = 110) frequently or always accepted drug samples, 39% always or frequently accepted stationery and 37% frequently or occasionally took part in sponsored CME (see Table 1).

**Table 1:** Physician-reported data on the frequency (percentage values in brackets) with which doctors (n = number of reporting physicians for each question) accepted remuneration for interviews, participation in sponsored CME and gifts when they were offered.

	Payments for interviews(n = 159)	Office Stati-onery(n = 159)	Day-to-Day items(n = 156)	Drug samples(n = 159)	Dinner invitations(n = 159)	Sponsored CME(n = 157)	Other*(n = 136)
Never	126 (79)	9 (5.7)	42 (26.9)	9 (5.7)	88 (55.3)	62 (39.5)	120 (88.2)
Rarely	24 (15 )	30 (19)	63 (40.4)	10 (6.3)	55 (34.6)	37 (23.6)	13 (9.6)
Occasionally	9 (6)	58 (36)	44 (28.2)	30 (18.9)	16 (10.1)	50 (31.8)	2 (1.5)
Frequently	0	51 (32)	5 (3.2)	80 (50.3)	0	8 (5.1)	1 (0.7)
Always	0	11 (7)	2 (1.3)	30 (18.9)	0	0	0

*e.g. textbooks, CME invitations without sponsorship, bottle of wine.

### Perceptions of the contact with the PSRs

43.3% (n = 68) of the doctors felt that they always (3.8%, n = 6) or frequently (39.5%, n = 62) received adequate and accurate information from PSRs, while 56% of the doctors believed that this was only occasionally (35.0%, n = 55), rarely (15.3%, n = 24) or never (6.4%, n = 10) the case. In response to the question about whether the prescribing behaviour of the individual doctors was influenced by the PSR, 6.4% (n = 10) believed that this was frequently the case, while 15.9% (n = 25) believed that this never happened. 35.7% (n = 56) believed that they were occasionally influenced and 42% (n = 66) thought that their prescribing habits were rarely influenced. If the two questions are considered together, it is apparent that there is no connection between the reported influence on prescribing behaviour and assessment of the accuracy of the information. Of the 67 doctors who felt that they always or frequently received adequate and correct information, 31 (46%) admitted that their prescribing habits were frequently or occasionally influenced. In contrast, of the 89 doctors who felt that they only occasionally, rarely or never received adequate and correct information, 35 (39%) admitted that their prescribing habits were frequently or occasionally influenced (Chi-square/Fisher exact test: p = 0.42).

### Impact of visits and perceptions on prescribing habits

Medical practices visited daily or 2–3 times per week by PSRs had a significantly higher number of prescriptions (mean ± SD; 11,308±4,963 vs. 8,912±5,721; p = 0.005, d = 0.456) and daily dose totals (mean ± SD; 639,602±292,409 vs. 311,358±491,374; p = 0.003, d = 0.87), but they did not have a higher total expenditure (mean ± SD; €470,534± €235,715 vs. €455,664± €330,345; p = 0.115, d = 0.054) than practices visited less frequently. However, as drug prescriptions and expenditure in medical practices are always dependent on the number of patients treated, we calculated the impact of the frequency of PSR visits on the corresponding factors in consideration of the number of patients. This showed that the number of prescriptions, the daily doses and the expenditure per patient were not higher at the frequently visited practices than at the more rarely visited practices, neither in total nor in the subcategories of on-patent branded drugs, off-patent branded drugs and generics (Table 2).

**Table 2:** Influence of the estimated frequency of PSR visits and the attitudes to these visits (subjective assessment of the adequacy of the information and influence on prescribing behaviour) on the number of prescriptions, the daily doses (defined daily dose, DDD) and expenditure per patient (means +/− SD).

		Average number of prescriptions perpatient during the assessment period			Average expenditure (€)per patient during theassessment period			Average DDD (mg) per patientduring the assessmentperiod		
		*On-patent* *branded drugs*	Off-patentbrandeddrugs	*Generics*	*On-patent branded* *drugs*	Off-patentbrandeddrugs	*Generics*	*On-patent* *branded drugs*	Off-patentbrandeddrugs	*Generics*
Frequency of visits	Daily. 2–3/week(N = 100)	1.2±0.5	0.7±0.3	5.4±1.7	201.7±287.6	37.4±37.1	126.6±36.8	71.5±31.6	34.2±14.7	356.9±143.0
	Stat. difference	*p = .449*	*p = .193*	*p = .241*	*p = .639*	*p = *.296	*p = .614*	*p = .537*	*p = .138*	*p = .215*
	1/week. 2/month. Less.Never (N = 58)	1.1±0.5	0.6±0.3	5.1±1.9	267.5±374.4	36.6±38.1	123.6±35.7	68.2±34.6	30.5±15.6	327.6±142.4
Perceived adequacyof information	Always. Frequently(N = 68)	1.3±0.5	0.7±0.3	5.1±1.7	243.1±267.0	43.8±38.2	125.1±34.7	74.8±40.1	34.7±15.3	318.0±132.3
	Stat. difference	*p = .044*	*p = .027*	*p = .206*	*p = .083*	***p = .005***	*p = .825*	*p = .149*	*p = .185*	*p = .027*
	Occasionally. Rarely.Never (N = 89)	1.1±0.4	0.6±0.3	5.4±1.9	202.6±340.3	31.2±34.8	126.4±37.7	67.1±25.9	31.4±15.1	368.4±146.6
Self-estimatedinfluence	Frequently.Occasionally(N = 66)	1.2±0.5	0.7±0.3	5.2±1.7	219.4±302.9	36.3±26.5	121.9±32.9	76.7±38.5	34.7±14.8	340.1±139.8
	Stat. difference	*p = .146*	*p = .068*	*p = .476*	*p = .194*	*p = .204*	*p = .308*	*p = .041*	*p = .175*	*p = .604*
	Rarely. Never(N = 91)	1.1±0.5	0.6±0.3	5.4±1.9	225.5±331.4	36.3±41.0	127.9±38.6	65.9±27.5	31.3±15.3	352.0±144.0

The p-values result from two-sided tests comparing the means reported above and below. Listed are all the significance levels of the differences. Differences nominally significant at a level of p≤0.005 (see methods) are reported with a bold font p-value.

With regard to the subjective assessment of to what extent the doctors felt that they received adequate and accurate information from the PSR, we divided the doctors into 2 groups: those who felt that they always or frequently received adequate and accurate information (n = 68), and those who felt that this was only occasionally, rarely or never the case (n = 89). As shown in Table 2, the doctors who felt that they always or frequently received correct information had higher expenditure on off-patent branded drugs per patient (mean ± SD; €43.82±38.23 vs. €31.25±34.76; p = 0.005, d = 0.347) than doctors who rated the adequacy of the information lower. In addition, the first group of doctors prescribed a lower proportion of generics (mean ± SD; 76.48%±11.60% vs. 81.39%±11.41%; p<0.005, d = 0.427) than doctors who rated the adequacy of the information lower.

With regard to the subjective assessment of how often the doctors felt that they were influenced by the PSR, we divided the doctors into 2 groups: one group that felt frequently or occasionally influenced (n = 66), and one group that felt rarely or never influenced (n = 91). There was no significant impact on the number of prescriptions per patient, the daily doses prescribed or on the expenditure per patient (see Table 2).

Table 3 shows the association of the frequency at which the doctors accepted gifts with the number of prescriptions, the daily doses, and expenditure per patient (the prescriptions, expenditures, and DDD for total drugs are not shown). The 62 doctors who always or frequently accepted office stationery prescribed higher daily dose totals per patient (mean ± SD; 491.97±158.95 vs. 420.53±140.57; p = 0.003, d = 0.483) and more generics (mean ± SD; 385.52±147.52 vs. 319.43±133.69; p = 0.004, d = 0.475; Table 3) than the 97 doctors who only occasionally, rarely or never accepted stationery. The 62 doctors who stated that they never took part in sponsored CME had a lower number of on patent-branded drug prescriptions per patient (mean ± SD; 1.05±0.35 vs. 1.27±0.55; p = 0.005, d = 0.457; Table 3), a lower proportion of on patent-branded drugs (mean ± SD; 40.74±13.72% vs. 47.64±16.58%; p = 0.001, d = 0.445), a higher proportion of generics (mean ± SD; 83.28±7.77% vs. 76.34±13.58%; p<0.0005, d = 0.596) and lower expenditure on off-patent branded drugs per patient (mean ± SD; €27.36±23.23 vs. €43.75±43.22; p = 0.002, d = 0.447; Table 3) in comparison to the 95 doctors who frequently, occasionally or rarely took part in such events.

**Table 3:** Influence of the frequency at which the doctors accepted gifts on the number of prescriptions, the daily doses (defined daily dose, DDD) and expenditure per patient (means +/− SD).

		Average number of prescriptions per patient during the assessment period			Average expenditure (€) per patient during the assessment period			Average DDD (mg) per patient during the assessment period		
		*On-patent* *branded* *drugs*	Off-patentbrandeddrugs	*Generics*	*On-patent branded drugs*	Off-patent branded drugs	*Generics*	*On-patent* *branded* *drugs*	Off-patentbrandeddrugs	*Generics*
Payments forinterviews	Occasionally. Rarely (N = 33)	1.3±0.7	0.8±0.2	5.4±1.6	210.9±281.0	38.2±35.6	126.5±40.0	79.3±47.1	38.1±14.9	358.4±134.6
	Stat. difference	*p = *.063	*p = *.065	*p = .690*	*p = *.815	*p = *.360	*p = .861*	*p = *.079	*p = *.024	*p = .551*
	Never (N = 126)	1.1±0.4	0.7±0.3	5.2±1.9	230.7±332.4	36.9±37.8	125.3±35.3	68.0±27.6	31.5±14.9	385.5±147.5
Officestationery	Always. Frequently (N = 62)	1.2±0.4	0.7±0.2	5.7±1.7	185.2±216.8	38.8±34.6	134.3±36.7	70.0±24.3	36.5±14.9	385.5±147.5
	Stat. difference	*p = *.891	*p = *.025	*p = .008*	*p = *.419	*p = *.049	*p = .014*	*p = *.910	*p = *.015	*p = * ***0.004***
	Occasionally. Rarely (N = 97)	1.2±0.5	0.6±0.3	5.0±1.8	253.1±372.4	36.1±39.0	119.9±34.9	70.6±37.3	30.5±14.8	319.4±133.7
Objects	Always. Frequently. Occasionally. Rarely (N = 114)	1.2±0.5	0.7±0.3	5.4±1.8	206.4±234.4	37.4±34.1	129±37.8	72.1±34.7	33.3±15.3	352.9±148.4
	Stat. difference	*p = *.448	*p = *.736	*p = .291*	*p = *.834	*p = *.406	*p = .047*	*p = *.248	*p = *.477	*p = .192*
	Never (N = 42)	1.1±0.5	0.7±0.3	5.0±1.7	260.2±461.1	35.5±43.6	123.8±37.2	65.2±27.2	31.4±14.5	319.8±114.3
Drugsamples	Always (N = 30)	1.2±0.4	0.7±0.2	5.7±1.4	159.6±124.2	30.0±14.1	132.9±31.2	75.0±23.6	33.4±13.2	387.2±129.8
	Stat. difference	*p = *.501	*p = *.477	*p = *.069	*p = *.607	*p = *.794	*p = *.217	*p = *.395	*p = *.821	*p = *.073
	Frequently. Occasionally. Rarely. Never (N = 129)	1.2±0.5	0.7±0.3	5.2±1.9	242.2±350.7	38.8±40.6	123.8±37.2	69.3±34.5	32.7±15.6	335.4±144.0
Dinnerinvitations	Occasionally. Rarely (N = 71)	1.2±0.4	0.7±0.2	5.2±1.8	214.3±260.6	40.6±35.6	125.5±38.5	73.1±27.7	35.8±14.2	346.9±147.8
	Stat. difference	*p = *.743	*p = *.058	*p = *.674	*p = *.428	*p = *.018	*p = *.988	*p = *.335	*p = *.025	*p = *.900
	Never (N = 88)	1.2±0.6	0.6±0.3	5.3±1.8	236.6±364.8	34.4±38.5	125.6±34.5	68.1±36.3	30.4±15.5	380.1±143.0
SponsoredCME	Frequently. Occasionally. Rarely (N = 95)	1.3±0.5	0.7±0.3	5.0±1.7	274.6±385.2	43.8±43.2	123.5±37.8	75.3±36.7	33.9±15.0	323.5±139.5
	Stat. difference	***p = *** **.005**	*p = *.183	*p = *.017	*p = *.025	***p = *** **.002**	*p = .*292	*p = *.022	*p = *.260	*p = *.015
	Never (N = 62)	1.1±0.3	0.6±0.3	5.7±1.8	157.4±174.4	27.4±23.2	129.7±33.6	63.1±24.4	31.1±15.3	380.1±143.0

The p-values result from two-sided tests comparing the means reported above and below. Differences nominally significant at a level of p≤0.005 (see methods) are reported with a bold font p-value.

## Discussion

This preliminary study is the first German study to show that there is an association between the frequency of PSR visits as estimated by doctors and their attitudes to these visits, and objective prescription figures. As already described in an earlier study [2], this study once again shows that there is close contact between doctors and PSRs and that only a small percentage of doctors accept no gifts from PSRs. It also shows that less than half of the doctors are convinced that they receive adequate and accurate information from PSR, and also that less than half believe that their prescribing habits are occasionally or frequently influenced by PSR visits.

In this study, we have investigated whether the common subjective feeling of being influenced is also associated with an objective change in prescribing data. The study provides preliminary evidence that the subjective perception that one is receiving adequate and accurate information about medicinal products, the acceptance of office stationery and the attendance at sponsored CME events are all associated with changes in objective prescribing data, while the frequency of the PSR visits, the self-perceived level of influence by PSRs and the acceptance of all other gifts are not associated with prescribing habits.

The results relating to the frequency of PSR visits and the acceptance of gifts from the pharmaceutical companies are largely consistent with the results of an earlier study by our research team [2]. The results are also largely in line with data from the USA collected in 2004. In their survey of doctors in the US in 2004, Campbell et al. showed that 94% of doctors accepted gifts from pharmaceutical companies [8]. This is largely in line with the data from our study, in which we found that only 3% of doctors accepted no gifts of any kind. A follow-up study by the same authors in 2009 showed that 84% of the doctors accepted gifts; this is still a high percentage, considering that contact between doctors and PSRs is subject to much critical discussion in the USA and in light of the many statutory regulations governing the sector [9]. In contrast, it is noticeable that doctors in the US accept dinner invitations much more frequently than their colleagues in Germany do [8], [9]. The perceptions of the doctors, namely the extent to which they feel that they receive adequate information from PSRs and/or whether they believe that they are influenced by them, are also relatively consistent with our earlier data [2], thus underlining the consistency of our findings. The observation that doctors find it harder to perceive the influence on themselves than the influence on their colleagues illustrates the frequently-mentioned “blind spot” that is associated with the topic of conflicts of interest; the existence of this “blind spot” makes it rather hard to change behaviour ([2]; see also [10], [11]).

In their systematic review, Spurling et al. (2010) found that, with a few exceptions, many studies have shown that contacts between doctors and pharmaceutical companies are associated with more frequent prescriptions, higher expenditure and lower prescription quality [5]. We also found – as others ([5], [12], but not all [13]) - that doctors in practices that are visited more frequently by PSRs more often prescribe drugs and prescribe them in higher daily doses. It is unclear whether the frequent visits are responsible for this, as it is also possible that large practices with high prescription figures are more likely to be visited by PSRs than small practices [14]. However, in monetary terms, the effects are irrelevant, as we did not find that the frequency of PSR visits had any effect on the total expenditure of the practices. If the patient numbers at a practice are taken into consideration, the significant effects of the frequency of PSR visits on prescribing behaviour were found to disappear, sometimes even completely.

With regard to the acceptance of gifts and their influence on prescribing data, we found that only the acceptance of office stationery and the attendance at sponsored CME events had an impact. The impact of the receipt of office stationery on prescribed total daily doses and generics was striking. If confirmed in larger studies, the association found in our study is a further argument against the frequently-voiced assumption that advertisements in the form of office stationery have no impact on doctors and underlines the existence of a relevant “gift relationship” already fostered by the acceptance of small gifts [15]. The fact that participation in CME activities relating to specific medicinal products has a positive effect on subsequent prescription of the advertised drug has been demonstrated in several (but not all) studies (systematic review in [5]). For example, participation in scientific symposia hosted in hotels at the expense of manufacturers [16] or participation in industry-sponsored CME courses [5], [17] increases the prescription rates of the advertised products, and close pharmaceutical contacts increase the likelihood that doctors will campaign for medicinal products from the corresponding manufacturers to be added to hospital drugs lists [18]. We were interested in the question of whether doctors who do not participate in sponsored CME have different prescribing data to doctors who do participate in such sponsored events. This study showed that the doctors who never took part in sponsored events had a lower number of prescriptions of on-patent branded drugs, a lower proportion of on-patent-branded drugs, a higher proportion of generics and a lower expenditure on off-patent branded drugs per patient. This underlines the influence of sponsored CME on general prescribing data and leads us to the tentative conclusion that promoting independent CME would probably change the prescribing behaviour of doctors [19].

We also investigated the effect of the doctors’ attitudes towards contact with PSRs on their prescribing habits. We found an association with changed prescribing data when we looked at the subjective impression of the adequacy and accuracy of the information provided by the PSR: Doctors who felt that they always or frequently received accurate information more often prescribed off-patent branded drugs per patient and prescribed a lower proportion of generics. It is therefore obvious that the belief that one is being supplied with accurate information is associated with changes in prescribing behaviour, even for medicinal products that are frequently advertised, namely off-patent branded drugs. This result extends recent findings from Canada, France and the United States demonstrating that physicians judged the quality of scientific information to be good or excellent in a similar percentage of promotions as in our study (54%) and indicated readiness to prescribe 64% of the time [20].

The study has several limitations that must be taken into consideration when interpreting the data: The choice of doctors and the low response rate of 11.5% (with the respective low power of the study) does not allow general statements to be made about all doctors and limits the representative nature of the statements. However, there is no reason to think non-respondents would be less likely to interact with PSRs. In fact, there may be even some underestimation of the effects since non-respondents may feel guilty about their contacts with PSRs and thus did not respond. Furthermore, we only have subjective estimates of the frequency of contacts and the acceptance of gifts and no objective data. With regard to sponsored CME, we also only asked about subjective assessments, and there was no objective data about whether the courses attended by the doctors were actually sponsored or not. Although physicians’ assessments of sponsorship are likely to be correct, there may be sponsoring of CME that they were unaware of. Therefore, assessments are likely to be an underestimate, which makes the finding even stronger. Furthermore, the outcomes used in this study do not necessarily show all impacts of promotion. Promotional strategies aim mainly to raise uptake of new drugs. But the indicators chosen in our study (on- and off-patent branded drugs or generics) may not be sensitive enough to show the impact on, for example, the uptake of new antidiabetic drugs as first-line treatments for patients with diabetes. In addition, the data give no indication about the quality of treatment: It is certainly conceivable that more prescriptions are accompanied by a better quality of treatment. We cannot comment on this. However, Spurling et al. (2010) show that contacts with pharmaceutical companies are associated with lower prescription quality on the part of the doctors [5]. Finally, we have not recorded or taken into consideration any other factors that could influence the prescribing habits of doctors and may interact with the PSR visits.

### Conclusion

These preliminary data provide evidence that the acceptance of office stationery, participation in sponsored CME and the belief that one is receiving adequate information from a PSR are associated with changes in the general prescribing habits of doctors. Further studies with larger sample numbers are required to confirm the data collected and to analyse interrelations with other factors influencing the prescribing habits of doctors.
